# Commentary: The atherogenic index of plasma is associated with an increased risk of diabetes in non-obese adults: a cohort study

**DOI:** 10.3389/fendo.2025.1605942

**Published:** 2025-07-17

**Authors:** Nahui Samanta Nájera-Segura, Hector A. Cabrera-Fuentes

**Affiliations:** ^1^ Universidad Nacional Autónoma de México (UNAM) - Universidad Autónoma Benito Juárez de Oaxaca (UABJO) Research Center, Faculty of Medicine and Surgery, Universidad Autónoma “Benito Juárez” de Oaxaca, Oaxaca, Mexico; ^2^ División de Investigación y Desarrollo Científico, Benemérita Universidad de Oaxaca, Oaxaca, Mexico; ^3^ R&D Group, Vice Presidency for Scientific Research and Innovation, Imam Abdulrahman Bin Faisal University, Dammam, Saudi Arabia; ^4^ División de Estudios de Posgrado e Investigación, Tecnológico Nacional de México, Instituto Tecnológico de Tijuana, Tijuana, Baja California, Mexico

**Keywords:** atherogenic index of plasma (AIP), metabolic risk, type 2 diabetes mellitus (T2DM), body mass index (BMI), predictive biomarkers, screening strategies, metabolic syndrome, clinical applicability

In this commentary, we analyze the findings of Cao et al. (2025) (1) and their implications for redefining metabolic risk assessment in non-obese adults. We focus on the Atherogenic Index of Plasma (AIP) as a valuable predictive tool for metabolic risk, its clinical applicability, and the critical need for further research to address current gaps in knowledge. The study highlights a crucial aspect of metabolic health—the risk of type 2 diabetes mellitus (T2DM) in individuals who, despite not meeting the BMI criteria for obesity, may still present with elevated metabolic risk factors.

## Expanding the role of AIP in people without obesity

Body Mass Index (BMI) is commonly used to evaluate metabolic risk, but it is clear that BMI alone does not capture the full picture. Many individuals classified as underweight or normal weight still develop metabolic disorders, suggesting the need for additional markers. Although the present commentary focuses on non-obese individuals, it is essential to acknowledge that AIP has also demonstrated strong associations with metabolic dysfunction in overweight and obese populations. Li et al. (2024), in a cohort of over 40,000 Chinese adults with elevated BMI, reported a J-shaped relationship between AIP and type 2 diabetes, with inflammatory mediators such as neutrophils and monocytes partially explaining the observed risk gradient (2). Complementarily, Karimpour Reyhan et al. (2024) identified AIP as the most accurate lipid index for detecting overweight and obesity in patients with T2DM, outperforming conventional lipid ratios with an AUC of 0.770 (3). These findings underscore the broader applicability of AIP as a biomarker of cardiometabolic risk, irrespective of adiposity status. Large-scale studies have further demonstrated that AIP is a strong predictor of metabolic risk even in non-obese individuals. Huang et al. (2024) analyzed data from over 85,000 normoglycemic adults with BMI <25 kg/m² and found a significant non-linear association between the TG/HDL-C ratio—a surrogate for AIP—and incident diabetes, with an inflection point at 1.36 (4). Similarly, Wu et al. (2022) reported a robust association between AIP and prediabetes in non-obese individuals with normal LDL-C levels, identifying a clinically relevant threshold at 1.617 (5). These findings support broader evidence indicating a non-linear relationship between AIP and diabetes risk, suggesting that even in the absence of obesity, elevated AIP levels may strongly predict increased predisposition to developing T2DM (1). However, the exact physiological mechanisms underlying this association remain to be fully elucidated. Given that AIP primarily reflects the triglyceride-to-HDL-C ratio, it is likely that chronic dyslipidemia, low-grade inflammation, and insulin resistance contribute to the observed link with diabetes (6, 7). Further exploration of these pathways is necessary to determine whether AIP is simply a surrogate marker or an independent metabolic risk factor. A recent cohort study further supports this association, demonstrating that higher AIP levels predict long-term diabetes risk even among individuals with normal fasting plasma glucose (8). The new consensus published by *The Lancet Diabetes & Endocrinology* (2025) advocates for a shift away from BMI-centric definitions of obesity, emphasizing metabolic dysfunction as a key diagnostic criterion (9). Despite this growing consensus, there remains significant debate on the best markers to replace BMI in clinical practice. Some researchers argue that waist-to-hip ratio, visceral fat quantification, and insulin resistance indices may offer more robust predictive value than AIP alone (10, 11). Comparative studies between these markers and AIP would be valuable to determine the optimal screening approach.

## Clinical implications and integration into practice

Cao et al. (2025) (1) identified a non-linear association between AIP and diabetes risk, with a threshold at AIP = -0.268. Individuals with AIP values above this point had a significantly higher risk of developing diabetes (HR 1.763, 95% CI: 1.210-2.568, *p* = 0.003) (1). This suggests the potential value of incorporating AIP into standard blood test panels and combining it with established tools such as the Framingham Risk Score. A summary comparison of AIP and traditional lipid markers is presented in **Table 1**.

**Table 1:** Summary of atherogenic index of plasma (AIP) findings and clinical implications.

Marker	Threshold/Association	Clinical Implication	Compared to Traditional Markers
AIP	> -0.268 (1); TG/HDL-C > 1.36 (4); AIP = 1.617 (5); ≤ 0.03 predicts prediabetes (19)	Predicts diabetes and prediabetes risk in both non-obese and obese adults; may reflect underlying insulin resistance and inflammation	Integrates TG and HDL-C; outperforms traditional lipid ratios in some T2DM populations (AUC 0.770); associated with metabolic dysfunction even within normal lipid ranges
LDL-C	Elevated levels increase CVD risk	Routinely used in lipid panels	Limited for detecting early metabolic abnormalities
HDL-C	Low HDL-C increases metabolic risk	Used independently and in AIP calculation	Alone may not capture full lipid-related risk
Triglycerides	Elevated TG linked with insulin resistance	Important component of metabolic panels	Incorporated into AIP for greater predictive insight

Summarizes current findings on AIP thresholds and its predictive utility across various BMI strata. AIP has demonstrated robust associations with diabetes, metabolic syndrome, and prediabetes, even in individuals with normal LDL-C and BMI values.

Compared to traditional lipid markers like LDL-C, HDL-C, and triglycerides, AIP integrates the ratio between triglycerides and HDL-C, potentially offering more nuanced insight into lipid-driven metabolic risk (12). Although its predictive potential is promising, several limitations must be addressed before clinical adoption. One key concern is the lack of standardization in AIP cut-off values across different populations (13). Given that lipid profiles vary by ethnicity, dietary habits, and genetic background, a one-size-fits-all approach may not be appropriate (14, 15). Furthermore, the stability of AIP over time has not been well characterized—fluctuations in triglyceride and HDL-C levels due to acute illness, dietary changes, or medication use could impact AIP’s reliability. AIP has also been linked to insulin resistance and beta-cell dysfunction, two key mechanisms in T2DM development (12, 16). This supports the notion that AIP is more than just a lipid ratio; it may reflect deeper metabolic disturbances related to lipid metabolism and inflammation. However, its added predictive value over existing risk models such as HOMA-IR (17) or the triglyceride-glucose index (18) remains unclear. Large-scale comparative studies are needed to determine whether AIP offers a distinct advantage or simply mirrors existing markers. Current evidence suggests that AIP is directly associated with prediabetes development, although the relationship appears to be non-linear. Zheng et al. (2023) found that AIP values ≤ 0.03 were significantly associated with increased risk of prediabetes (HR = 1.90; 95% CI: 1.66–2.16; p < 0.0001), whereas higher values did not show a significant association (19). Additionally, Cai et al. (2024) used trajectory modeling to show that individuals with persistently elevated AIP levels had a higher risk of progressing from prediabetes to diabetes (20). These findings suggest that AIP may serve as an early indicator of dysglycemia, reflecting subtle metabolic changes before overt hyperglycemia develops.

## Critical considerations and future research directions

### Ethnic and Geographic Validation

Given that the study was conducted within a Chinese cohort, it is imperative to validate these findings in diverse ethnic and geographic populations. This will ensure that AIP’s predictive value is universally applicable and not limited to a specific demographic. Emerging data suggest that lipid metabolism and diabetes risk differ across ethnic groups, and applying a single threshold universally may lead to misclassification in certain populations.

### Establishment of standardized cut-off values

The absence of universally accepted AIP thresholds limits its clinical integration. A global consensus on standardized cut-off values would greatly enhance AIP’s reliability and consistency in clinical practice.

### Clarification of mechanistic pathways

Further research is essential to determine whether AIP directly contributes to metabolic dysfunction or if it serves as a reliable proxy for underlying disturbances in lipid metabolism and inflammation. Understanding its mechanistic role will clarify its utility as a clinical tool. For instance, does AIP correlate with markers of systemic inflammation such as C-reactive protein (CRP), or with early indicators of endothelial dysfunction? Exploring these relationships could provide mechanistic insights. The association between AIP and type 2 diabetes in individuals with obesity is likely mediated by its reflection of lipid abnormalities commonly seen in insulin-resistant states. Gong et al. (2024) demonstrated that AIP was a strong, independent predictor of metabolic syndrome in adults with T2DM, with a nonlinear relationship and an AUC of 0.840 (21). Furthermore, a systematic review by Andraschko et al. (2025) confirmed elevated AIP levels in individuals with metabolic syndrome, including those with T2DM, across diverse populations (22). However, to date, no robust studies have evaluated the role of AIP in individuals with type 1 diabetes, highlighting a critical gap in the literature that warrants further investigation.

### Comparative evaluation with established biomarkers

To fully assess AIP’s value, direct comparisons with traditional lipid markers such as LDL-C, HDL-C, and triglycerides are crucial. Such studies will help define its added predictive value and determine whether AIP offers distinct advantages in identifying metabolic risks.

### Optimization of study designs

Future research should prioritize well-designed longitudinal cohort studies and randomized controlled trials to establish causal relationships and strengthen the evidence supporting AIP’s role in metabolic risk prediction. Moreover, interventional studies evaluating whether targeted modifications of AIP—through lifestyle interventions or pharmacological treatments—can effectively reduce diabetes incidence would provide essential insights into its potential as a modifiable risk factor.

### Identification of high-risk populations

It is crucial to conduct targeted research focusing on non-obese individuals with metabolic syndrome, as well as underrepresented demographics, including adolescents and older adults. Such studies will help refine risk stratification models and provide deeper insights into how AIP can be applied to diverse populations, ultimately improving its clinical utility in broader contexts.

Addressing these critical gaps will be pivotal in establishing AIP as a reliable and accessible tool for metabolic risk assessment.

## Conclusion

The study by Cao et al. (2025) (1) significantly advances the recognition of AIP as a valuable metabolic risk marker that goes beyond traditional BMI classifications. By elucidating AIP’s non-linear relationship with diabetes risk, this research offers important insights into refining screening strategies, particularly for individuals who may be overlooked by BMI alone. Recent studies have extended these findings, showing that AIP is also a strong predictor of metabolic dysfunction in overweight and obese adults, highlighting its utility across the full adiposity spectrum. Despite this promise, standardized cut-off values and broader population validation are needed before clinical adoption. Evidence also suggests AIP may serve as an early indicator of dysglycemia and disease progression, warranting further investigation through prospective and interventional studies.The 2025 Lancet (9) consensus further underscores the necessity of moving beyond BMI-centric approaches, advocating for the integration of biomarker s, and AIP may play a complementary role alongside indices like HOMA-IR or the TyG index. AIP holds potential as a valuable addition to metabolic risk assessment, but its role must be carefully contextualized within the broader landscape of predictive biomarkers. Future research should aim to define its place among other lipid indices and determine whether it improves upon existing risk stratification tools. By addressing these research gaps, we can refine metabolic risk assessment strategies and improve early identification of individuals at risk for T2DM, ultimately enhancing preventive healthcare strategies.
